# Lipidomics Issues on Human Positive ssRNA Virus Infection: An Update

**DOI:** 10.3390/metabo10090356

**Published:** 2020-08-31

**Authors:** David Balgoma, Luis Gil-de-Gómez, Olimpio Montero

**Affiliations:** 1 Analytical Pharmaceutical Chemistry, Department of Medicinal Chemistry, Uppsala University, Husarg. 3, 75123 Uppsala, Sweden; david.balgoma@ilk.uu.se; 2 Center of Childhood Cancer Center, Children’s Hospital of Philadelphia, Colket Translational Research Center, 3501 Civic Center Blvd, Philadelphia, PA 19104, USA; gildegomel@email.chop.edu; 3 Spanish National Research Council (CSIC), Boecillo’s Technological Park Bureau, Av. Francisco Vallés 8, 47151 Boecillo, Spain

**Keywords:** lipidomics, ssRNA+ virus, membrane fusion, lipid metabolism, cholesterol, sphingolipids, phosphatidylinositol, SARS-CoV

## Abstract

The pathogenic mechanisms underlying the Biology and Biochemistry of viral infections are known to depend on the lipid metabolism of infected cells. From a lipidomics viewpoint, there are a variety of mechanisms involving virus infection that encompass virus entry, the disturbance of host cell lipid metabolism, and the role played by diverse lipids in regard to the infection effectiveness. All these aspects have currently been tackled separately as independent issues and focused on the function of proteins. Here, we review the role of cholesterol and other lipids in ssRNA+ infection.

## 1. Introduction

The ongoing COVID-19 pandemic is developing (July 2020) worldwide with devastating global consequences, both for social organization and healthcare systems. COVID-19 illness is brought about by infection with the severe acute respiratory syndrome coronavirus SARS-CoV-2 [1,2], which is an enveloped positive single-stranded RNA virus (ssRNA+) [3]. The most abundant studies related to human diseases induced by ssRNA-positive viruses refer to *Picornaviridae*, *Coronaviridae*, and *Flaviviridae* [4].

This impact in a short time span has brought the Biology and Biochemistry of viral infection mechanisms to reach momentum. The infection mechanisms have been described for diverse unrelated viral families [5], with the majority of them being DNA viruses. Within *Picornaviridae*, *Coronaviridae*, and *Flaviviridae*, Rhino and Poliovirus (Picornaviridae), SARS-CoV, Middle East Respiratory Syndrome Coronavirus (MERS-CoV), Hepatitis C virus (HCV), West Nile virus (WNV) and Dengue virus (DENV) fall within the viruses whose life cycle biology is better known. Nonetheless, knowledge regarding virus entry mechanisms and other related features of the virus life cycle has been gained from the research on the influenza virus from the *Orthomysoviridae* family and the human immunodeficiency virus from the *Retroviridae* family. Consequently, these and other unrelated viruses will be also considered in this review from the point of view of the different aspects that affect the lipidomics of the viral infection.

All ssRNA+ viruses initially infect mammal cells through the interaction of virus proteins with any given host cell protein. Further fusion of the virus and host cell membranes is required for the viral genetic material to get into the cell. Once inside the cell, the genomic and subgenomic viral RNAs are translated into the virus proteins; these then lead the virus replication, which is a process that involves modulation of the host cell lipid metabolism [3,5,6]. Consequently, along with other features, current lipid studies about the aforementioned virus infection focus their research on membrane fusion and modulation of the lipid metabolism of the host cell. These two processes are considered separated disciplines of the infection.

The fight against the virus infection encompasses primarily the inhibition of the binding of the viral spike protein to the host cell’s receptor protein. Consequently, most of the current research focuses on the role played by viral proteins but the lipid environment, where the proteins carry out their function and regulation, is considered secondarily [7]. Nevertheless, improving the knowledge on how the lipids are involved in the mechanisms of infection may provide clues to develop treatments and better counteract the virus-induced pathology [3]. To fill this gap, here, we review the main aspects regarding the lipidome regulation of the viral infection mechanism by ssRNA+ viruses.

## 2. Virus Entry: Lipid Rafts and Membrane Domains

### 2.1. Membrane Mechanical Properties Required for Virus Infection

The initial step in virus infection is the binding of any viral structural glycoprotein to a receptor of the host cell. The spike protein accounts for such function in coronaviruses (CoVs) and other enveloped viruses. After the virus is attached to the host cell protein, the process of membrane fusion starts to get the viral genome into the host cell. This process implies viral envelope and host cell membrane fusion, for which an energetically cost-effective barrier must be overcome. For example, in coronavirus, membrane fusion is driven by the fusion peptide (class I), which is localized within the spike protein (S protein) and becomes active after cleavage of the S protein at specific sites by host proteases or pH-dependent mechanisms [4,6,8]. A different mechanism of attachment and endocytosis drives the virus entry in the case of HCV. This mechanism is more complex than that of coronaviruses and involves interaction of the virus envelope E1 and E2 proteins (class II fusion loop) with several host cell proteins [9,10,11]. However, a membrane fusion-driven pore is also required in HCV to deliver the viral genetic material into the host cell cytoplasm.

Two main mechanisms of membrane fusion have been described: viral endocytosis by host cell membrane (endocytic pathway), and both viral and host cell plasma membrane fusion (non-endocytic pathway). After docking of the virus to the attachment factor or the receptor on the host cell surface, the virus may internalize its genomic material or the entire particle [12,13,14]. The non-endocytic pathway encompasses the direct delivery of the genetic material through a pore formed in the cell membrane by the induction of viral proteins at neutral pH. This pathway is typical of non-enveloped viruses. The endocytic pathway is more complex and harnesses the host cell endocytosis machinery for the virus internalization. Three main ways have been described in the endocytic pathway, namely: the clathrin-mediated endocytosis (CME), the caveolae-mediated endocytosis (CavME), and the macropycnocytosis. The best-known endocytic mechanism is the clathrin-mediated endocytosis. The CME is used by small to intermediate-sized viruses. This mechanism uses vesicles coated by the protein clathrin, which forms a polyhedral lattice that surrounds the cell membrane-derived vesicle where the virus is internalized into the cell cytoplasm through the early endosomes. Clathrin coating is coordinated by the adaptor protein (AP-2) and other adaptors; it is less commonly AP-independent. The protein dynamin is involved in regulating the clathrin-coated vesicle (CCV) formation as well as its scission from the membrane. Some viruses proceed to membrane fusion at this stage for releasing their genome into the cytoplasm. The early endosomes have a pH of about 6.0 to 6.5; therefore, it is considered that membrane fusion is not strictly pH-dependent. Other viruses need a lower pH for the membrane fusion to be effective; thus, it is considered pH-dependent. A further step leading to endosome maturation to become late endosomes with a pH of about 5 has to proceed before the membrane fusion takes place and the genetic material is delivered to the cytoplasm. Sequential acidification of the virus proteins from the early to late endosomes has also been suggested through the self-organized endosomal network. Maturation of the early endosomes to late endosomes and trafficking between them is controled by the Rab proteins, which are members of the Ras superfamily of small G proteins. Subsets of Rab proteins differ between the early and late endosomes, and the Rab subset change is accompanied for by formation of the phosphoinositide PI(3,5)P_2_ from the precursor PI(3)P. Regarding lipid composition, early endosome membrane lipids are primarily composed of unsaturated and short alkyl chains, whereas long and saturated alkyl chains, such as in gangliosides, are predominant in the membrane lipids of late endosmes. Membrane fusion in some viruses requires a further step in which late endosomes are fused with lysosomes, this step giving rise to the late endosome/lysosome pathway. Cholesterol depletion driven by its synthesis inhibition or extracting agents as methyl-β-cyclodextran (MβCD) is used to assess whether the virus entry takes place through the caveolae/raft endocytosis. This pathway in less known and encompasses the formation of initial endocytic vesicles enriched in cholesterol from lipid-rafts, with complex signaling routes that involve the activity of tyrosine kinases and phosphatases. Thereafter, the cargo is transported to the endoplasmic reticulum (ER) through early and late endosomes. Most of the viruses using this endocytic pathway have different gangliosides as receptors, mainly GM1, which has a high concentration in caveolae. Polyomavirus, which are non-enveloped DNA viruses that replicate in the nucleus, use preferently this endocytic pathway, but picornaviruses and the coronavirus HCoV-229E have also been reported to internalize through the Caveolae-mediated endocytosis [15]. Macropinocytosis is a phagocytic-like mechanism of virus entry that is currently utilized by the cell to internalized fluids; it is dependent on actin and implies the actin cytoskeleton rearrangement to enable internalization of the virus particle [14]. Macropinocytic vacuoles (macropinosomes) are formed after membrane ruffles fold to reach at its end the membrane again, and the vacuole is closed through self-membrane fusion. These vacuoles containing the viral particle may traffick afterwards through the early and late endosome network. Macropinocytosis is common to large-sized viruses. However, recent work [16] has shown that Ebola virus (EBOV) may use a macropinocytosis-like process to entry the host cell in a clathrin, caveolae, and dynamin-independent manner, but dependent of actin and a lipid raft. Conversely, this virus may use as well an endocytic pathway that is dependent on clathrin, caveolae, and dynamin. Which endocytic route is used by this virus depends on the host cell type. Description of the current methodologies used to study the entry route by viruses can be found in reference [14].

Some viruses may use different entry mechanisms, this feature being likely dependent upon the membrane lipid composition of the host cell they infect as well as the particular cell surface factor attachment used. CME is the entry route currently used by HCV, HIV-1, EBOV, rotaviruses, and some coronaviruses, even though other routes can also be used as for EBOV (see above). A reaction between clathrin and actin seems to be necessary for the effective entry of these viruses. Regulation by microtubules of the CME has been reported for flaviviruses. DENV, WNV, and Semliki Forest Virus (SFV, Alphavirus family, *Togaviridae*) have been found to depend on early endosomes (Rab5 protein marker) for entry but not late endosomes (Rab7 protein marker), which means that they do not have strict low pH requirements or depend on different acidification mechanisms for membrane fusion. Conversely, influenza avian virus (IAV) needs both early and late endosomes to entry, thus reflecting low pH dependence for membrane fusion. Marburg virus (MARV) may use for internalization a CME through the endo/lysosomal pathway. Coronaviruses differ in their internalization mechanism among strains. Thus, while HCoV-229E is known to use the Cav-ME route, SARS-CoVs use an endocytic pathway that is clathrin- and caveolae-independent but receptor and pH-sensitive, with lipid rafts playing an essential role [17]. This endocytic mechanism implies internalization of the receptor protein angiotensin-convering enzyme 2 (ACE2) along with the spike protein into the early endosomes, but the receptor is afterward recycled to the membrane via lysosomes. Nonetheless, previous studies showed that SARS-CoV could enter through a pH-independent direct membrane fusion as it could infect cells that do not express ACE2, such as enterocytes and hepatocytes [18]. Recent research on the virus SARS-CoV-2 points to pH-independent direct cell and viral membrane fusion, which is a process that is driven by the subunit S2 of the spike protein after cleavage by the cellular serine protease TMPRSS2 [19]. On the contrary, the infectious bronchitis virus (IBV), a gamma-coronavirus, was reported to use the CME pathway to entry, with vesicle scission being mediated by GTPase dynamin 1, and a dependence on low pH and lipid raft localization of the receptor. Tracking of the virus trip inside the cell was followed by using diverse inhibitors, cholesterol sequestering agents, and virus particles labeled with fluorescent markers. Membrane fusion takes place at the late endosome/lysosome step of the endocytic pathway, with deep rearrangement of the host cell cytoskeleton being induced by the endosomal viral cargo [15]. Accordingly, viruses may sequester on their own profit the diverse endocytic pathways that are currently used by the host cell, but variability of the proteins and even the general mechanisms may also exist as a consequence of virus specifity.

Membrane fusion has been described to proceed through the catalytic action of three different types of fusion peptides or fusion loops of class I, II, or III. These proteins afford the free energy necessary to overcome through conformational changes the kinetic barrier due to repulsive hydration strength. Most of the knowledge on the viral and host membrane fusion has been gained from the influenza virus and its type I fusion peptide hemagglutinin. A detailed description of the three fusion peptide-guided mechanisms involved in membrane fusion has been previously reviewed in [20,21,22]. Bringing the viral and the host membranes closer enough (c.a. 20 Å) for inducing the membrane fusion is a process that entails membrane curvature and changes in the lipid bilayer phase. They are driven by the insertion of a hydrophobic region of the fusion peptide, which requires dehydration of the inter-membrane space. Nonetheless, from experiments with no-protein fusogens, such as polyethilen glycol, it seems that membrane curvature stabilization is not a key player in membrane pore opening. The calculated displacement of lipids in the outer leaflet of the host membrane accounts for no more than 10% of the membrane area (about 3500 Å^2^), which does not represent a substantial energetic demand [21]. This energetic burden has been demonstrated to be afforded by the cooperation of three fusion peptides in influenza virus membrane fusion [23], whereas two adjacent trimers of the fusion protein are required in West Nile virus [24]. This result points to the fact that the viral membrane curvature may not actually impose a constraint for proceeding to the hemifusion step and the formation of a steep curvature stalk, where the outer leafleats are merged. By the mesurement of electron density profiles through X-Ray reflectivity in stalks formed from bilayers in a lamellar state with different lipid compositions, Aeffner et al. [25] determined that the inter-bilayer separation should attain 9.0 ± 0.5 Å in order to facilitate dehydration and promote stalk formation. These authors also found that increasing the relative proportion of nonbilayer-forming, cone-shaped lipids, such as glycerophosphoethanolamine or cholesterol, favored the stalk formation by reducing the hydration energy barrier and, possibly, by contributing with their intrinsic negative curvature. As well, the energy required for dehydration was, in this study, found to decrease with the length of the acyl chains of the glycerophospholipids. However, the hemifusion stalk stage was not detected by Gui et al. [26] using fluorescence and electron microscopy. The results of this study show that such a stage might be an unstable intermediate that is quickly resolved toward the postfusion stage. Contrarily, localized point-like contacts were abundantly visualized in this study, where the dimples formed in the target membrane, about 5 nm wide, were drawn toward the virus surface. They were able to detect up to well-resolved four types of virus–target membrane contacts at pH 5.5 and 5.25 using liposomes of dioleylglycerophosphocholine, DOPC, with 20% cholesterol. At the lowest pH, a tight contact of the two membranes through an extended length of about 100 nm (catalogued by the authors as type III) was the predominant interaction, whose abundance was increased by about 3-fold in cholesterol-containing liposomes in comparison to only DOPC liposomes.

Using synthetic peptides that resemble the fusion peptide hemagglutinin and electron spin resonance (ESR), Ge and Freed [27] found that the most relevant effect of the synthetic fusion peptides was the induction of highly ordered membrane domains, which came motivated by virtue of electrostatic interactions between the peptide and negatively charged phospholipid headgroups. A similar effect was reported for two putative fusion peptides enclosed in the spike glycoprotein of SARS-CoV-1. It was found in this study that the inner water content in the lipid bilayer was dropped by the insertion of the fusion peptide as a consequence of increased lipid packing, but only in membranes containing negatively charged lipids, whereas the water content was only slightly altered in zwitterionic dipalmitoylglycerophosphocholine (DPPC) liposomes [28]. Additionally, the fusion peptides created opposing curvature stresses in the highly bended membranes containing nonbilayer-forming phospholipids. However, previous studies had pointed out that interaction with the lipid headgroups is not an essential factor in reaching the membrane hemifusion state [21,29]. In SARS-CoV, the possibility of existing two fusion peptides that act in coordination has been suggested [7]; one of the peptides would promote the dehydration process, while the other one would act in modifying/disturbing the lipid organization within the target membrane [26,28,30]. Hence, the catalytic role of the fusion peptide(s) is likely to tackle three properties of the target membrane in the virus entry machinery: (i) dehydration of the intermembrane space for the fusing membranes coming into the required proximity, (ii) to promote negative curvature to form the hemifusion stalk, and (iii) to alter the lipid packing density, which will be generated in the highly curved local dimples of the stalk [22,28]. The effectiveness of these three processes is likely to depend upon the membrane lipid composition. Further research is devoted to this issue, and new clues are expected to come from electron and fluorescence microscopy [31].

### 2.2. Raft Lipids Related to Virus Entry

**Figure 1:**
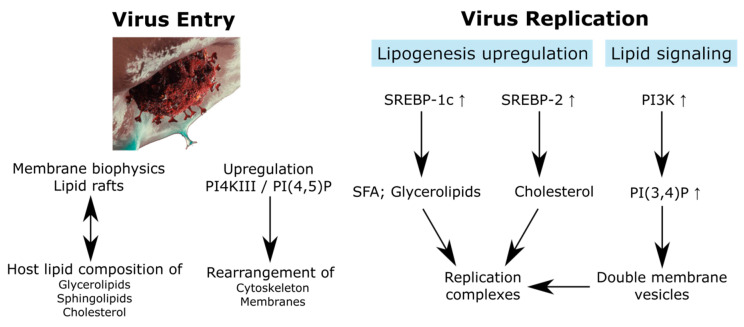
Relationship between the virus entry and replication with the lipidome. SREBP, sterol regulatory element binding protein; SFA, saturated fatty acid. SARS-CoV-2 artwork was modified from a work from *We Are Covert*, who allows anyone to use it for any purpose including unrestricted redistribution, commercial use, and modification.

Since the dominant phospholipid in the outer leaflet of most membranes is the bilayer-forming, positive charged diacylglycerophosphosphocholine (PC), the idea was raised that the viral docking to the receptor on the target cell and, consequently, the membrane fusion were likely to take place at specific microdomains with particular lipid composition, the so-called lipid rafts [27,32,33,34,35,36]. A special characteristic of the lipid rafts is the high content of cholesterol [37,38,39]. Even though a high content of sphingolipids and gangliosides is also a defining characteristic of lipid rafts (Figure 1), direct in vivo visualization still remains unresolved [39].

An unexplored possibility is that rafts do not have a permanent localized existence, but they arise under the induction of certain proteins such as the hydrophobic insert of the viral fusion peptide or the fusion loop. This fact might be also responsible for bringing negatively charged lipids from the inner leaflet of the bilayer to its outer leaflet by flip-flop mechanisms. This hypothesis would explain the promotion of virus entry by the interaction of the fusion peptide with the negatively charged phospholipid headgroups [25,27] as well as the kinetics of the membrane fusion [25]. A number of studies have shown that the hemifusion step and pore widening are sped up after increasing the relative concentration of cholesterol in the bilayer composition, whereas either the depletion of cholesterol in the cell culture medium or the inhibition of cholesterol synthesis by statins was able to halt the viral infection at the virus entry step [26,27,28,40,41]. The effect of cholesterol on promoting membrane merging has also been observed for Bis-(monoacylglycero)-phosphate (BMP) [26]. This particular phospholipid was shown to be strictly necessary for Dengue virus (DENV) entry even at low endosomal pH [42]. As pointed out above, the exact role played by cholesterol is not known in detail, but its intrinsic negative curvature seems to be an essential characteristic in promoting the stalk formation during virus entry. However, a recent study shows that the cholesterol action is likely to involve a direct influence on the oligomeric state of the fusion peptide after insertion into the host cell membrane, as well as on the effects of the fusion peptide on the membrane reorganization and dynamics [43]. In another recent study, a new lipid-label-free methodology was used to measure the kinetics of influenza virus infection [44]. According to the results of this study, cholesterol is able to augment the efficiency of membrane fusion in a receptor binding-independent manner. Nevertheless, the rate of membrane fusion was not altered. These results led the authors to conclude that the positive effect of cholesterol in membrane lipid mixing is related to its capability to induce negative curvature. Since membrane mixing was achieved in this latter study without binding of the spike protein of the influenza virus to the host cell receptor, the catalytic effect of the fusion peptide might proceed in an independent way in this virus. Cleavage of the spike protein in SARS-CoV-1 does not seem to be also necessary for the fusion peptide to become fusogenic, but rearrangement of disulfide bridges in the S1 peptide after receptor binding are likely involved in the conformational changes driving the fusion mechanism [43,45]. Contrary to these latter results, which point to the fact that membrane fusion is independent of viral protein attachment to its receptor, Guo et al. reported lipid raft-dependent viral protein binding with the suppression of viral infection if the lipid rafts were disrupted with cholesterol drug-induced depletion; lipid rafts, as recognized by the caveolin-1 marker, were the membrane domain where structural proteins of the infectious bronchitis virus (IBV) co-localized but the nonstructural proteins did not [35]. The question regarding whether the lipid-raft domains may serve as platforms to concentrate the proteins required for viral entry and, even though some evidence exists, to activate signaling pathways inside the host cell still remains unsolved.

**Figure 2:**
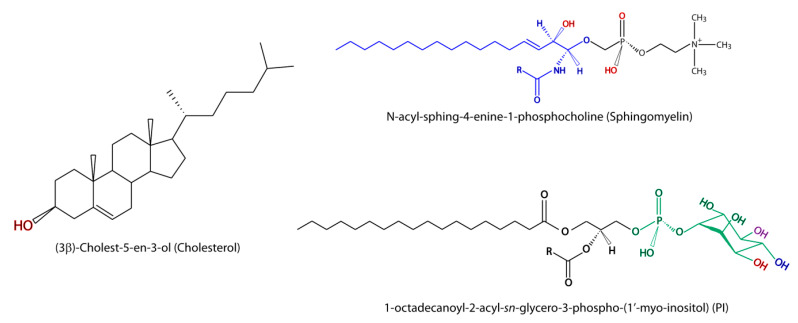
Structure of the most relevant lipids in virus infection is illustrated. Hydroxyl (HO) and oxygen (O) atoms potentially involved in the interaction with the fusion peptide or fusion loop are marked in red in cholesterol and sphingomyelin. The basic ceramide structure is marked in blue in the sphigomyelin structure. In phosphatidylinositol (PI), the hydroxyl groups that can be esterified with phosphate at the positions 3, 4, and 5 of the myo-inositol group to render PIP (PI3P or PI4P), PIP2 (PI(3,4)P or PI(4,5)P), and PIP3 (PI(3,4,5)P), which are marked in red, blue, and violet, respectively, are shown.

Sphingomyelins (SMs) are also common lipids found in lipid rafts, which contribute to make these membrane microdomains detergent-resistant [34]. The structure of a representative of this lipid class is illustrated in Figure 2. The ganglioside GM1, a sphingolipid, is used as a marker of lipid rafts [34]. Sphingolipids (SLs) promote to an extent higher than Chol the liquid-ordered phase in the outer leaflet of the membrane bilayer because of the long saturated acyl chains they currently contain (the R group in Figure 2 may extend to a length of up to 26 C), in addition to their capability to form intermolecular hydrogen bonds [46]. A relevant function of the lipid rafts has been suggested to be the connection between the events outside the cell with the pathways inside the cell, thus acting as ‘signaling platforms’. With the aim of this function to be properly accomplished, the lipid rafts would act as concentrators of specific transmembrane proteins, mainly receptors, whose compatibility with the membrane phase would determine their selectivity. Thus, SLs would account for a role in connecting the outer leaflet with the inner leaflet through their long saturated acyl chains. Regarding virus entry, research has been primarily focused toward the role played by cholesterol, but a number of studies have also enlightened the SM influence on this early step of viral infection. The displacement of cholesterol by SMs and the other way round has been demonstrated, with the bilayer liquid-ordered phase being preferentially determined by the interaction between SM and cholesterol. This interaction would be controlled to a certain extent by the intracellular actin meshwork, which would also be responsible for the compartmentalization of the membrane into lipid-specific domains [47]. Furthermore, the actin role is possibly extended to the routing of the viral genomic material toward the replication place inside the host cell. The hydrolysis of SM by sphingomyelinases to render the corresponding ceramide in specific membrane domains is proposed to regulate the dynamics of cholesterol in the cell membrane, the effect of such regulation being the progressive disassembly of cholesterol from the liquid-ordered phase and its displacement. Since the interaction of ceramides with cholesterol has been suggested to be an apoptotic regulator, it can be expected that viral proteins would act in recruiting cholesterol to displace the ceramide and to avoid the programmed cell death. This fact is added to the other characteristics conferred by cholesterol to the membrane mechanical properties discussed above. To study the influence of ceramide on membrane fusion during Semliki Forest Virus (SFV, Alphavirus family, *Togaviridae*) infection, ceramide analogs have been used [48]. According to this experiment, in which cholesterol-containing PC plus PE liposomes were used, the roles played by the 3-hydroxyl group and the 4,5-*trans* carbon-carbon double bond of the sphingosine backbone (Figure 2) were found to be essential in the fusion process. In additon, ceramide was the simplest SL to accomplish this significant contribution in mediating the fusion, independently of the length of the acyl chain. More recently, a Ca^2+^-dependent pathway of infection by the Rubella virus (RuV, Rubivirus family, *Togaviridae*) was demonstrated to proceed through direct binding of the fusion loop in the viral E1 protein to SM/cholesterol-enriched membranes [49]. However, the treatment of host cells with sphingomyelinase proved that SM is exclusively required for viral entry but is not required for the further steps of viral replication. SM in the host cell membrane and acid sphingomyelinase (ASMase) activity have also been shown to be required by the Ebola virus (EBOV), a negative single-stranded RNA virus belonging to the *Filoviridae* family, to get into the host cell. The ASMase activity renders ceramide that provoques raft enlargement and membrane invagination [50]. This study also showed that the virus was able to recruit both SM and ASMase to the raft where the viral attachment was happening. Conversely, Bovine herpesvirus 1 (BoHV-1, *Herpesviridae* family) seems to require SM in the virus envelope but does not in the host cell [51]. The role played by ceramides is contradictory as they may enhance or inhibit virus replication, but this SL action seems to be related to the viral replication phase rather than to the internalization phase [52,53,54]. In virus using the endocytic pathway, similar to the influenza virus or the Ebola virus, it has been shown that activity of glucosylceramidase (GBA) is required for viral entry and membrane fusion through the regulation of endocytosis, but in a virus-dependent manner. It was also shown that trafficking of the epidermal growth factor (EGF) to late endosomes was impaired in GBA-knockout cells, which negatively affects the virus entry through spoiling the endocytic pathway [55]. Indeed, co-clustering of the HA attachment factor and EGF in submicrometer domains that overlap partially has been reported recently [56]. Accordingly, there is evidence that SLs have a function in enveloped ssRNA viruses at the early stage of infection that accounts for the viral entry modulation, but further research is still necessary to unveil the exact mechanisms of SL reactions.

Some CoVs (HCoV-OC43 and HCoVHKU1), as well as influenza A virus (whose fusion loop is hemagglutinin, HA) and other non-related viruses (i.e., non-enveloped simian virus 40 SV-40, of polyomavirus family), use the sialoglycan moiety (9-*O*-acetyl-sialic acid) of gangliosides or glycoproteins located in membrane lipid rafts as receptors for the spike protein. The amino acid Trp90 in the domain A of the HCoV-OC43 S protein was shown to be essential for receptor binding. However, despite the fact that binding to 9-*O*-acetyl-sialic acid is required for membrane fusion, further interaction of the virus protein with other host membrane sialoglycans or proteins is also necessary to induce the conformational changes leading to membrane fusion [57,58]. Conversely, formation of the complex SV40 protein with the host cell ganglioside GM1 was found to be enough to induce the membrane curvature and invaginations required for membrane fusion [59].

As already discussed above, some studies have depicted the possibility that interaction of the fusion peptide or fusion loop with negatively charged phospholipids on the host membrane might be required for an efficient membrane fusion [25]. In this regard, phosphatidylserine (PS) contained in the virus envelope has been demonstrated to serve after externalization as a virus co-receptor through the T cell immunoglobulin mucin domain 1 (TIM-1) receptor in EBOV and other viruses, even in an indispensable fashion [60,61,62,63]. In the study of Nanbo et al. [63], flipping of PS from the inner leaflet to the outer leaflet of the cell membrane for virion adquisition and incorporation to its envelope is proposed as a previous step to TIM1 binding. In herpes simplex virus (HSV), phospholipid scramblase-1 (PLSCR1), after activation by HSV exposure, flips both PS and Akt to the outside of the membrane in a Ca^2+^-dependent mechanism. PS is restored to the inner leaflet 2 to 4 h after infection to avoid apoptotic triggering [62], suggesting a different role for PS in relation to the TIM-1 PS receptor. However, the function of TIM-1 as an essential receptor for HAV has been disputed [64] due to the finding that quasi-enveloped HA virions (eHAV) were able to infect TIM1-knockout Vero cells to a similar extent to naked HAV. Hence, the authors proposed TIM1 to be an accessory attachment factor by binding PS on the HAV envelope rather than an essential virus protein receptor. In spite of these contradictory data, PS seems to act in any way in virus attachment and entry in certain virus families, at least contributing to the process efficiency, but the exact role may depend on every virus or it may be complementary to other factors.

A phospholipid currently associated to the inner leaflet of the lipid rafts is phosphatidylinositol (PI), which is a negatively charged phospholipid with important and versatil signaling functions (Figure 2) [65,66]. Abundant data suggest that a derivative of PI, the phosphatidylinositol 4,5-biphosphate (PIP2), accumulates preferently in liquid-disordered phases (L_d_) [7], where the cholesterol content is presumed to be low, and interplays with PS, which is rather localized in liquid-ordered phases (L_o_). PIs play an essential role also in endosome maturation, which is a requisite for efficient virus infection of those using the endosomal pathway [56,66]. During HIV infection, PIP2 has been proposed to coordinate the actin cytoskeleton changes required for efficient virus entry in CD4+ T cells [67]; after virus attachment to the host cell receptor, PIP2 is recruited to the binding membrane microdomain, and in this way, PIP2 controls the protein reactions, leading to actin polymerization. As well in HIV-1, the requisite of PIP2 accumulation for the virus Gag protein to be properly anchored and stabilized in the inner leaflet of the cell plasma membrane has been pointed out [68,69]. Two isoforms, α and γ, of the phosphatidylinositol-4-phosphate 5-kinase family type 1 (PIP5K1) have recently been shown to participate in Gag stabilization by PIP2 through targeting the Gag precursor Pr55^Gag^ to the cell plasma membrane [70]. As commented above, interaction with the headgroup of negatively charged phospholipids such as PS or PI may also contribute to the dehydration process in the formation of the hemifusion stalk, with this contribution happening by promotion of the inverted hexagonal phase in the lipid bilayer and binding of Ca^2+^ [25]. In in vitro experiments with COS-7 cells and multilamellar vesicles (MLVs), unspecific binding of the Marburg virus (MARV) mVP40 protein to PIP, PIP2, and even PIP3 species present in the MLVs, both in the presence or absence of PS, has been reported. In this study, it was also found that with increasing PS concentration, the association of mVP40 to MLVs rose up to a threshold. Furthermore, the addition of sphingosine with the aim to reduce the negative charge load in the inner leaftet of the COS-7 cells led to a decrease in the binding level. These facts suggest that the electronic density, rather than the specific lipid species, is a determinant factor for binding [70]. Activation of the PI3K pathway for signaling is one of the most relevant features taking place for both entry and budding during infection by a number of viruses [58,71,72,73]. PI3K converts PIP2 into phosphatidylinositol 3,4,5-triphosphate (PIP3). In addition to stabilizing proteins or serving as a binding factor, PIP2 has been shown to collaborate with Akt through the signaling pathway PI3K/Akt on avoiding apoptotic events, and in this way, keeping the host cell metabolically active for virus replication and budding [71,72,73].

All these results clearly bring evidence that the lipid environment surrounding proteins involved in virus infection has a relevant function in the virus entry mechanism. Different lipids are essential for virus docking to the cell receptor either serving directly as (co)-receptors or providing the appropriate environment (lipid rafts) for the necessary reactions (e.g., membrane curvature). In addition, the virus, through specific protein conformational changes, takes advantage of several cell signaling pathways controlled by diverse membrane lipids. This process allows the virus to govern the cell metabolism following endocytosis of the viral genetic molecules.

## 3. Lipid Regulation in Virus Replication: Viral Factories

After the virus or its genome gets inside the infected cell, ssRNA+ viruses and other enveloped ones that replicate in the cytoplasm manage the cell metabolism to develop the replication scaffold, this membrane structure bolstering the so-called ‘virus factory’ [5,58,74,75,76,77,78,79,80]. There is consensus on that the functions of these structures are (i) to compartmentalize the diverse processes involved in viral genome replication, its envelopment, and structural protein assembly; (ii) to increase virion concentration during budding before infecting naïve cells; and (iii) to create a protected environment to escape the innate immune recognition of the viral components. Virus replication imposes an extra-energetic expenditure to the cell metabolism. Hence, cell central metabolism is orchestated by viral proteins to redirect toward the generation of enough energy and metabolites that are required for virus replication. In particular, building the scaffold demands a high rate of new lipid synthesis. Therefore, the lipid metabolism is hijacked by the virus proteins for the *de novo* synthesis of fatty acids in order to generate the scaffold membranes, the replication complexes (RCs), as well as for energy production in the β-oxidation pathway in the mitochondria. Concurrently, the cell metabolism needs to be kept above a threshold level to avoid exhaustion of the host cell. Full understanding of the mechanisms and related factors involved in virus–host interaction is a requisite for developing efficient antiviral infection therapies.

### 3.1. Viral Replication Complexes

The scaffold structure raised for building the viral factory varies between different virus in their morphology and possibly lipid composition. Flaviviruses develop a so-called ‘membranous network’ (MN) in a spherule/invagination type, while coronavirus does through a quarter-like type delimited by ‘double membrane vesicles’ (DMVs). Nonetheless, HCV (*Flaviviridae*) uses DMVs instead [77]; hence, this morphological separation may have exceptions or be somewhat diffuse. An extended review of the different virus family-related morphologies of the MNs as well as diverse factors influencing their formation can be found in [22,75,76]. It should be remarked that the exact lipid composition of the RCs’ membranes is not known in detail yet, although there is evidence that their lipid profile differs from that of the organelles from which they are generated. The enrichment of typical lipids such as cholesterol, sphingomyelins, and glycosphingolipids in the lipid rafts seems to be a common feature of these MNs. The RCs’ membranes may be originated from the endoplasmic reticulum (ER) in the perinuclear area, as for example in SARS-CoV and *Faviviridae* [75,78,80], from the Golgi, giving rise to cytopathic vesicles (CPVs) as in *Togaviridae* and *Picornaviridae* [75], from mitochondria (*Nodaviridae*) [79], or from the cell plasma membrane (CPVs in Alphaviruses) [75]. However, vesicle trafficking between the ER and the Golgi organelles may contribute to an undefinition in this regard. MNs, and in particular DMVs, are connected to the cytosol through a pore, which is believed to serve as the gate to the replication scaffold for the requiered metabolites, in particular nucleotides. This pore-mediated gate has not been detected up to date in SARS-CoV’s DMVs, which raises the concern of how the required metabolites get inside the RCs. There is evidence from a number of studies that DMVs are the site of replication, but it has also been shown that DMVs can be developed irrespective of whether RNA replication takes place by the sole action of the viral proteins, at least for HCV [81,82]. Viral nonstructural proteins nsp3, nsp4, and nsp6 are involved in DMV development in SARS-CoV-1 in a time-dependent manner and correlating with RNA replication. Timecourse events have been shown to run with the initial formation of single membrane vesicles (SMVs) during the first 2–4 h after cell infection. These futher evolve to DMVs 16 h after infection, and they ultimately turn into multimembraneous vesicles (MMVs) close to the *cis*-Golgi at the budding stage 36–48 h after infection, this latter transformation being coincident with the formation of vesicle packets [75,78,79,83]. In HCV, NS5A seems to be enough for DMV formation, but the collaboration of NS3-5B is required for completing efficient DMVs, whereas NS4B is likely responsible for inducing the formation of SMVs [77,80,82]. Even though particular hints can be likely associated to every particular virus, there are common features shared by all ssRNA+ viruses regarding RCs’ structure and buildup.

### 3.2. Lipid-Related Host Factors Associated to the RCs’ Buildup

Enveloped viruses such as ssRNA+ viruses have a membrane lipid whose profile is different to that of the original organelle membrane when the envelope is created. Since the viral membrane is known to be enriched in cholesterol, sphingolipids, and phospholipids with saturated acyl chains, the DMV is believed to be also primarily composed of such classes of lipids. An unusual sphingolipid, dehydrosphingomyelin, along with PS and plasmalogens of PE were reported in the HIV envelope [84]. A role for sphingomyelin-to-ceramide conversion has been proposed in WNV budding, as its envelope was found to be highly enriched in sphingomyelin [85]. More recently, using multi-color super-resolution microscopy and mass spectrometry analysis, a substantial increase in PIP2 (from 11% to 51%) and PIP3 (from 0.01% to 0.13%) was reported in the HIV membrane as compared with the plasma membrane of the host cell [69]; this fact is related to the recruitment of Gag protein for efficient membrane fusion as aforementioned (Figure 1).

However, the most striking and known lipid-related factor associated to the MNs’ development is the PI4KIII signaling pathway. The PI4Pα isoform, which is mainly expressed in the ER, has been shown to be a key factor for HCV replication, whereas the PI4KIIIβ is found in the Golgi and is required by Picornaviruses and some HCV strains [75]. This enzyme interacts with the viral protein NS5A, and disrupting this interaction prevents virus replication. The product of the PI4K enzyme is PIP4; enrichment in this PI has been shown to act in different processes regarding virus replication: membrane curvature, directly or indirectly through recluting cholesterol [86], glycosphingolipid transport to the RCs by the action of the FAPP2 protein [87], and protein concentration. However, conversely to these studies, it has been shown that currently used inhibitors of PI4KIIIα, enviroxime and BF738735, actually exert their inhibition against PI3K [88]. Thus, this result points out a genomic dependence on the PI kinases in HCV; otherwise, the action on PI3K is required only at the entry stage (see above). Enviroxime-like inhibitors have been shown to halt enterovirus replication through the action against PI4Kβ [89]. The *de novo* lipid synthesis has also been evidenced for WNV, from the *Flaviviridae* family as HCV, to proceed in a PI4P-independent fashion and, concurrently, it is not related to PI4KIII signaling [90]. There is no clear evidence on the fact that the PI4K signaling pathway has a relevant function in MNs’ development. Hence, while PI4KIIIβ was shown to be important for SARS-CoV’s DMV formation [91], another study did not find its metabolite, PI4P, within the host factors involved in SARS-CoV replication, and the authors attibute to PI4P a function rather in virus entry. However, the authors of this latter study acknowledge that siRNA methodology may provide false negatives [92,93]. Since DMVs are not common in healthy cells but they can be observed during authophagy, it has been suggested that SARS-CoV and other coronaviruses use the autophagy pathway for development of the DMVs; indeed, it has been shown that nsp6 in MHV or the equivalent nsp5-7 in arteriviruses, which hits the ER, can activate such a pathway [79,94]. Nonetheless, DMVs are smaller than autophagosomes, and hence, they might be rather endoplasmic reticulum derived vesicles (EDEsomes) enriched in PI3P and not follow exactly the same synthetic route [94]. Further work on coronaviruses and autopahgy found that only the LC3-I protein, the microtubule-associated proteins 1A/1B light chain 3B, is localized on the replication membranes, but the active protein lipidated with phosphatidylethanolamine LC3-II inserted into the autophagosome membrane is absent. Accordingly, present knowledge on coronaviruses in regard to autophagy suggests that they take benefit of the autophagocytic components but do not develop autophagosomes per se [95].

The autophatocytic pathway has also been associated to the start of HCV infection, but it seems not to be necessary for the infection to go on [82]. Later on, it was shown that autophagy was key in RNA replication at the onset of HCV infection [96], but the virus life cycle can go ahead afterward without the autophagy system intervention. Further work has shown that HCV, and possibly DENV, uses the autophagy system to evade the innate immune system [97]. Using immortalized human hepatocytes defective of the autophagy-related proteins either beclin (BCN1) or ATG7, it was shown in the latter study that disruption of the autophagy machinery elicites activation of the interferon signaling pathway and leads to apoptosis of the infected cells. Triggering of the autophagy pathways takes place after binding of the virus to the cell surface via the downregulation of mTOR and inactivation of Akt signaling [95]. Conflicting results have been reported for the induction of autophagy by HCV in regard to the unfolded protein response (UPR) [95]. Recent work [98] has bound the induction of autophagy by HCV to Golgi membrane fragmentation to render vesicles that colocalize with the HCV replicons. The immunity-related GTPase M protein (IRGM) mediates the phosphorylation of the early autophagy initiator ULK1 as well as the Golgi membrane fragmentation in response to HCV infection. The protein LC3 has also been detected in the replication membranes of the HIV-1, and the association of LC3-II with Gag-derived proteins seems to be a requisite for the efficient maturation of the Gag subunit p24 [14,99]. Members of the *Picornaviridae* family, non-enveloped viruses, have been reported to subvert the autophagosome pathway as a means to exit the infected cell without membrane lysis; support for this spreading mechanism comes from the finding of numerous extracelular vesicles that are enriched in phosphatidylserine phospholipids [14]. The best studied virus regarding autophagy is the dengue virus (DENV). Even though it was initially suggested that the DENV replication complexes are developed from autophagosomes, further work pointed out that the replication of DENV took place on invaginations arising from the endoplasmic reticulum (ER), while autophagy was rather used by DENV to modify the lipid metabolism in a way that is known as lipophagy [100,101]. Lipophagy was first shown to be an active way to get energy under starvation [102] through the association of autophagic components with lipid droplets (LDs). Recently, lipophagy has been demonstrated to regulate the fatty acid availability for the β-oxidation through contact sites between the mitochondria and the ER [103]. Regarding virus-associated hijacking of the cell lipid metabolism, Heaton and Randall [100] early showed that increased β-oxidation and the depletion of triglycerides was concurrent with and necessary for DENV replication. Then, these features were linked to the action of autophagy through the association with lipid droplets. A recent study by Zhang et al. [104] has found that AUP1, a type III protein with signals for LDs and ER, plays a relevant role in lipophagy induced by DENV and other flaviviruses such as WNV. Unmodified AUP1 is required for lipophagy triggering. A 10-fold increase in the content of diacylglycerophosphocholines (PCs) was measured in this study in infected cells containing unmodified AUP1, this increase being concomitant with a depletion of triacylglycerols and cholesterol esters, whereas the contents of free fatty acids and unesterified cholesterol rose. Conversely, smaller LDs, but not a reduction of their abundance, were observed in AUP1-knocked-out cells. Thus, these data point to an augmented consumption of LDs in the infected cells. This study unveils the mechanism that leads to the commented results; after the DENV protein NS4A associates with AUP1, the complex is relocalized from LDs to autophagosomes, where the acyltransferase domain of AUP1 is activated for the generation of phospholipids. This process was found to be dependent on the AUP1 ubiquitylation status, with NS4A inhibiting the ubiquitylation of AUP1.

Similar to viral entry, cholesterol has been found to be also relevant in the RCs’ membranes [79,82]. Up to a c.a. 9-fold enrichment of cholesterol was found in HCV-developed DMVs as compared to its content in the ER membranes from which DMVs were originated [77]. A key protein in cholesterol metabolism associated to non-vesicular transport is the oxysterol-binding protein (OSBP). This protein has been described to transport cholesterol to PI4P-enriched membranes, which would agree with its collaboration in delivering cholesterol to DMVs with an abundant content of this PI [77]. The ceramide transfer protein (CERT) and the four-phosphate adaptor protein 2 (FAPP2) are known to undergo a similar fate in HCV infection [82]. An important protein involved in cellular lipid homeostasis is the sterol regulatory element binding protein (SREBP), a bHLH-zip transcription factor with three isoforms; SREBP1c regulates the expression of fatty acid (FA) biosynthesis genes [105,106], whereas SREBP2 transactivates genes implied in cholesterol biosynthesis, intracellular lipid transport, and lipoprotein import [107]. A recent study shows that the inhibition of SREBP with the retinoid derivative and RAR-α agonist AM580 prevents MERS-CoV infection by avoiding the formation of functional DMVs [105]. In this study, the lipid metabolism was the most affected pathway, with sterol biosynthesis being strengthened at expense of the glycerophospholipid metabolic pathways. Fast activation of the lipid biosynthesis enzymes Acetyl-CoA carboxylase (ACC), fatty acid synthase (FAS), and HMG-CoA synthase (HMGCS) was observed in such study, whose activity was partially blocked by AM580 inhibition of SREBP enzymes. Promotion of lipid biosyntheis after infection had already been pointed out for HCV in an elegant proteomics and lipidomics study [108]. HCV infection elicites changes in the proteome of host cells that resembled the Warburg effect described in cancer cells toward lactate production and the support of continuous glycolysis; concurrently, the up-regulation of citrate synthase (CS) and other lipogenic enzymes 24 h after infection was interpreted by the authors of the latter study as indicative of re-routing of the tricarboxylic acid (TCA) cycle for cytosolic accumulation of citrate, which would be used in FA synthesis. The up-regulation of peroxisomal and mitochondrial FA oxidation pathways is concurrent with the other metabolic changes. An increase in pro-apoptotic ceramides was observed in the latter study as well; two possible interpretations were attributed to this finding, either a cytopathic effect after cell cycle arrest over time enough to complete virus offspring or a defense response of the host cell to avoid infection spread.

Blocking cholesterol suitability for the membraneous network or endosomes used for the virus replication and internalization has been demonstrated to inhibit the virus life cycle in a number of unrelated viruses. Disruption of the SREBP pathway restrains the Andes virus (ANDV), an ssRNA- virus, internalization, although it does not bind to the cell surface receptor [109]. In addition to SREBP2, other components of this pathway were found to be necessary. The dependence of viral entry on the sterol regulatory element binding protein cleavage activating protein (SCAP) and the site 1 protease (S1P) was evidenced in cells null for these proteins. Thus, in the study of Petersen et al. [109], the virus was not internalized in cells lacking S1P, this result pointing out that a complete cholesterol biosynthesis pathway is required. Infectivity was also reduced 10-fold when the cells were treated with methyl-β-cyclodextrin (MβCD), a cholesterol sequestering agent, and comparable results were obtained after cell treatment with mevastatin or the S1P inhibitor PF-429242. However, the S1P dependence of virus infectivity does not seem to affect other viruses, thus this route being likely selective for hantaviruses [110]. In this study, the genetic or pharmacological disruption of the SREBP pathway at the site of the regulatory element membrane-bound transcription factor peptidase/site 1 protesase (MBTPS1/S1P) dramatically reduced viral infection, which is a feature that confirms the essential dependence of hantavirus on the high membrane cholesterol content for membrane fusion and effective infection. The down-regulation of sterol synthesis at the gene level after infection was found to be controlled by an interferon regulatory loop, in which a type I interferon-dependent mechanism down-regulates the expression of SREBP2 [111], this result showing a link between the innate immune response and cholesterol biosynthesis after viral infection. This type I interferon response toward cholesterol synthesis down-regulation was dependent on the mevalonate-isoprenoid branch as a supply of mevalonate completely blocked the cholesterol synthesis, whereas a supply of cholesterol did not. Additionally, in the presence of geranylgeraniol, the type I interferon inhibition of sterol biosynthesis was severely diminished. Further research has shown that interferon may regulate the sterol synthesis pathway in multiple forms through microRNAs [112]. In particular, miR-342-5p was found to hit multiple SREBP-independent targets of the mevalonate–sterol synthesis pathway after viral infection. The type I interferon response was also observed in regard to the impairment of the formation of double membrane structures induced by arteriviruses as replication sites [113]. Host cell fight against viral infection by a reduction of cholesterol availability has been also pointed out to come from the antiviral effector protein interferon-inducible transmembrane protein 3 (IFITM3). This protein interacts with vesicle-membrane-protein-associated protein A (VAPA), impeding its association with the oxysterol binding protein (OSBP), and consequently, altering the normal function of OSBP. As a result of the IFITM3 action, virus release into the cytosol is blocked by the accumulation of cholesterol in multivesicular bodies and endosomes. This effect restrains the membrane fusion of the intraluminal vesicles and that of the multivesicular bodies, which is a requisite for virus budding and release to the cytosol [114]. The viral accesory protein of HIV Nef competes with the cholesterol transporter ABCA1 to prime the transport of cholesterol to lipid rafts as a viral strategy to raise the replication membranes, thus overcoming the antiviral properties of ABCA1 [115].

The replication of Rabies virus (RABV), an ssRNA virus, is halted by the action of viperin (virus inhibitory protein, endoplasmic reticulum-associated, IFN-inducible) in RAW264.7 cells. This protein is induced by the RABV, IFV, HIV, or HCV infection through promotion of the innate immune response bound to the TLR4 signaling pathway. The inhibitory activity of viperin on virus budding is related to its capability to substantially drop the contents of cholesterol and sphingomyelin in the replication membranes [116], thus pointing out the relevance of the membrane lipid composition for efficient virus replication. The induction of viperin has also been proven for HCV and IFAV [111]. However, viperin does not intervene in the inhibition of arterivirus-induced double membrane formation [113].

## 4. Additional Pathways of Lipid Metabolism Affected in Virus Infection

Remodeling of the lipid metabolism by virus infection may leave signals at the organism level even some years after healing. The metabolome profile of patients undergoing SARS-CoV-1 infection during the outbreak of 2002–2003 was assessed 12 years after overcoming the pathology [117]. An outstanding result of this study regarding disturbed lipid metabolism was the elevation of phosphatidylinositol (PI) and lysophosphatidylinositol (LPI) species concentrations in serum, which in turn correlated positively with the levels of very low-density lipoproteins (VLDL); higher concentrations of products of the phospholipase A_2_ (PLA_2_) such as lysophospholipids (LPPLs) and free arachidonic acid (AA) were also found in patients as compared to healthy voluntiers, with a correlation between the level of AA and the ratio of LPI(18:0) to total 18:0-PIs being observed as well. These results show a potential high sensitivity of SARS-CoV patients to PLA_2_ activity. In the general context, the metabolome of these patients pointed to hyperlipidemia, cardiovascular abnormalities, and glucose metabolism alteration as a delayed efffect of the viral infection. Nonetheless, the authors acknowledge that some of the related metabolic disturbations are likely owed to the pharmacological treatment. High levels of PLA_2_ group IID (PLA2G2D) in lungs of middle-aged mice as compared to young mice had previously been associated to a fatal or worse outcome [118]. The authors of this study conclude that the negative influence of this enzyme in SARS-CoV infection was to increase the concentration of anti-inflammatory lipid mediators, mainly protaglandin D_2_ (PGD_2_), which impaired the efficient function of the immune system [119]. In the recent SARS-CoV-2 outbreak (COVID-19), mortality has mostly affected aged people above 60 years old, thus showing an age-related fatality as for SARS-CoV-1 and MERS-CoV [120]. Using a lipidomics approach, the effect of HCoV-229E and MERS-CoV infection on the host cell lipid profile was recently investigated in cell culture [121]. The main conclusions of this study agree with the raised content of AA and LPPLs through PLase activity, which indicates that the possible virus-induced activation of cPLA_2_ favors virus replication as a factor required for DMVs’ formation. In this study, linoleic acid (LA) or AA supplementation to the culture cells suppressed replication, which is a result that may be interpreted as a demonstration of the perturbation of the LA/AA axis of the lipid metabolism.

In the COVID-19 outbreak, it has been suggested that increasing the levels of vitamin D could help fighting against the SARS-CoV infection [122]. This suggestion is based on the fact that 25-hydroxyvitamin D3 was found to protect Huh7 cells against MERS-CoV [105]. Vitamin D is a lipid-related compound belonging to the group of fat-soluble secosteroids, with the most important form in humans being vitamin D3 (cholecalciferol) [123]. In a recent study, high doses of vitamin D have shown protective effects against DENV infection through regulation of the Toll-like receptor expression as well as the modulation of pro-inflammatory cytokines release, which suggests that its action is focused toward the immune system modulation rather than to lipid metabolism [124]. However, evidence on the beneficial effects of vitamin D uptake is still poor, and more studies are devoted to this issue.

Lipids, as components of membranes, are related to viroporins, which are specific viral proteins that are known to create ion channels for ion trafficking [125,126,127]. The effect on cell metabolism of diverse viroporins differs among them, but there is evidence that they are closely related to viral pathogenity [125]. Viroporins may play a relevant role during virus infection, as they are involved in membrane permeability and calcium homeostasis. Their participation in the development of vacuoles from the ER during the DMVs’ formation has been suggested, but data on this issue are still scarce. The regulation of Ca^2+^ flux by viroporins might favor the membrane fusion through the interaction of this cation with the phospholipid headgroups and concurrently facilitate the required dehydratation reaction. Viroporins are not required for virus replication with the exception of rotaviruses and picornaviruses; thus, whether this function is exerted through the ion channels or another property of viroporins remains an issue still unknown [125]. The lipid composition of the membrane may influence the viroporin activity, leading to different versions of ion channels, which depends on the electric charge that the phospholipids confer to the membrane and curvature [127]. A viroporin from rotavirus, NSP4, was shown to co-localize with the autophagy marker protein LC3 in membranes accomodating virus replication; this viroporin is implicated in the sequestering of autophagy for the transport of proteins from the ER to the replication sites [128]. Further research is necessary to understand the role played by viroporins in virus infection in order to consider them as potential therapeutic targets.

## 5. Conclusions

**Figure 3:**
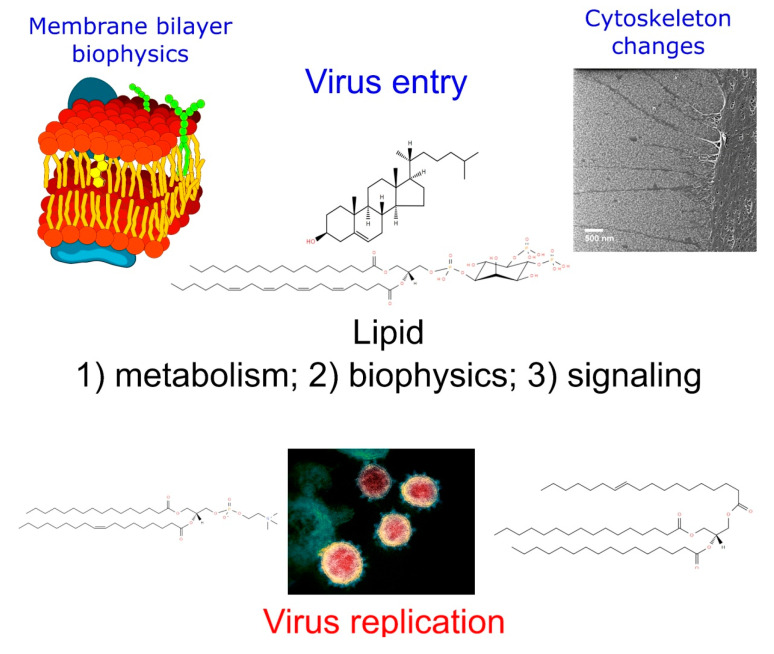
Comprehensive view of the virus replication process and the main lipids involved in every step.

Remodeling of the virus-induced host cell lipid metabolism is a remarkable feature of the viral infection that affects viral entry, replication of the genomic material, and the releasing of progeny. A comperhensive view of the process is illustrated in Figure 3. The main actors are well known to be cholesterol, sphingolipids, and PIs, but other lipid species and their related pathways such as the LA/AA axis are also relevant. How to target the lipid metabolism in a safe manner to avoid virus infection or reduce its pathogenity is a promising therapeutic tool, but it demands improving the knowledge on the actual pathways that are affected over the virus life cycle. The exact mechanism through which the enzyme inhibitors act on the key enzymes of the lipid metabolism is additionally required to develop more efficient and safe therapeutic drugs. Since the lipid metabolism is essential for proper cell function, selective drugs targeting the virus or exclusively the infected cells have to be used to avoid harmful side effects.
